# Effect of intra-arrest trans-nasal evaporative cooling in out-of-hospital cardiac arrest: a pooled individual participant data analysis

**DOI:** 10.1186/s13054-021-03583-9

**Published:** 2021-06-08

**Authors:** Fabio Silvio Taccone, Jacob Hollenberg, Sune Forsberg, Anatolij Truhlar, Martin Jonsson, Filippo Annoni, Dan Gryth, Mattias Ringh, Jerome Cuny, Hans-Jörg Busch, Jean-Louis Vincent, Leif Svensson, Per Nordberg, PRINCE, Maaret Castren, Frank Eichwede, Pierre Mols, Tilmann Schwab, Michel Vergnion, Christian Storm, Antonio Pesenti, Jan Pachl, Fabien Guerisse, Thomas Elste, Markus Roessler, Harald Fritz, Pieterjan Durnez, PRINCESS investigators, Patrick Goldstein, Nick Vermeersch, Adeline Higuet, Francisco Carmona Jiménes, Fernando Rosell Ortiz, Julia Williams, Didier Desruelles, Jacques Creteur, Emelie Dillenbeck, Caroline Busche, David Konrad, Johan Peterson

**Affiliations:** 1 Department of Intensive Care, Hopital Erasme, Université Libre de Bruxelles (ULB), Route de Lennik, 808, 1070 Bruxelles, Belgium; 2 Department of Medicine Center for Resuscitation Science, Karolinska Institute, Solna, Sweden; 3 Emergency Medical Services of the Hradec Kralove Region, Hradec Kralove University Hospital, Hradec Kralove, Czech Republic; 4 Emergency Department, SAMU Centre Hospitalier Régional Universitaire de Lille, Lille, France; 5 Department of Emergency Medicine, University Hospital of Freiburg, Faculty of Medicine, University of Freiburg, Freiburg, Germany

**Keywords:** Cardiac arrest, Intra-arrest, Hypothermia, Outcome, Randomized clinical trial

## Abstract

**Background:**

Randomized trials have shown that trans-nasal evaporative cooling initiated during CPR (i.e. intra-arrest) effectively lower core body temperature in out-of-hospital cardiac arrest patients. However, these trials may have been underpowered to detect significant differences in neurologic outcome, especially in patients with initial shockable rhythm.

**Methods:**

We conducted a post hoc pooled analysis of individual data from two randomized trials including 851 patients who eventually received the allocated intervention and with available outcome (“as-treated” analysis). Primary outcome was survival with favourable neurological outcome at hospital discharge (Cerebral Performance Category [CPC] of 1–2) according to the initial rhythm (shockable vs. non-shockable). Secondary outcomes included complete neurological recovery (CPC 1) at hospital discharge.

**Results:**

Among the 325 patients with initial shockable rhythms, favourable neurological outcome was observed in 54/158 (34.2%) patients in the intervention and 40/167 (24.0%) in the control group (RR 1.43 [confidence intervals, CIs 1.01–2.02]). Complete neurological recovery was observed in 40/158 (25.3%) in the intervention and 27/167 (16.2%) in the control group (RR 1.57 [CIs 1.01–2.42]). Among the 526 patients with initial non-shockable rhythms, favourable neurological outcome was in 10/259 (3.8%) in the intervention and 13/267 (4.9%) in the control group (RR 0.88 [CIs 0.52–1.29]; *p* = 0.67); survival and complete neurological recovery were also similar between groups. No significant benefit was observed for the intervention in the entire population.

**Conclusions:**

In this pooled analysis of individual data, intra-arrest cooling was associated with a significant increase in favourable neurological outcome in out-of-hospital cardiac arrest patients with initial shockable rhythms. Future studies are needed to confirm the potential benefits of this intervention in this subgroup of patients.

**Supplementary Information:**

The online version contains supplementary material available at 10.1186/s13054-021-03583-9.

## Introduction

Out-of-hospital cardiac arrest (OHCA) is one of the major health issues of the industrialized world [1]. Despite decades of efforts to promote cardiopulmonary resuscitation (CPR) education and the introduction of automated external defibrillators, less than 50% of cardiac arrest victims achieve return of spontaneous circulation (ROSC) and this percentage drops to 20% or less for those patients that live in rural areas or those with an initial non-shockable rhythm (i.e. asystole and pulseless electric activity) [2].

Post-anoxic brain injury remains the first cause of death in resuscitated cardiac arrest patients and there is a need to develop strategies to mitigate these injuries to improve neurologic outcome [3]. Therapeutic hypothermia (known more generally as targeted temperature management [TTM]) may reduce ischemic and reperfusion brain injury after cardiac arrest. In 2002, two randomized clinical trials (RCTs) demonstrated the benefit of TTM on favourable neurologically recovery in patients who were cooled in hospital for 12–24 h to 32–34 °C following an out-of-hospital cardiac arrest with an initial shockable rhythm (i.e. ventricular fibrillation or pulseless ventricular tachycardia) [4, 5]. Based on these studies, international guidelines recommended the use of TTM as routine treatment of OHCA patients with shockable rhythm and extended its use also to all cardiac arrest patients [6]. More recently, the clinical benefits from TTM were also demonstrated in non-shockable rhythms [7]. In addition, guidelines state that patients resuscitated from OHCA should be cooled as soon as possible; however, the optimal timing to initiate cooling procedures in this setting remains unknown [8, 9].

Experimental data suggested that hypothermia initiated during CPR (i.e. “intra-arrest cooling”) would provide additional beneficial effects against post-anoxic brain injury [10]. However, data from human studies are limited [10, 11] and inconclusive; moreover, not all methods to induce intra-arrest cooling may produce the same effects on brain recovery after cardiac arrest (CA). In one study, Bernard et al. showed that intra-arrest cooling using cold fluids was associated with a decrease in ROSC, in particular for patients with shockable rhythm, with no significant difference in survival at hospital discharge with the control group [12]. In a recent systematic review including two RCTs [13, 14], intra-arrest cooling using trans-nasal evaporative cooling was associated in two RCTs with a non-significant trend towards a higher proportion of OHCA patients achieving favourable neurologic outcome when compared to patients where TTM was initiated after hospital arrival [10]. Moreover, the benefits of this intervention were observed in particular among those patients with an initial shockable rhythm [13, 14], although this has been not been further evaluated.

As such, it remains unclear whether, according to the rhythm, intra-arrest cooling using trans-nasal evaporative cooling might be beneficial in this setting. The aim of this pooled analysis on individual participant data from two randomized studies, PRINCE [13] and PRINCESS [14], was to investigate the effects of intra-arrest trans-nasal evaporative cooling on neurologic outcome in OHCA patients with initial shockable or non-shockable rhythms.

## Methods

### Overview of the PRINCE and PRINCESS trials

PRINCE and PRINCESS were two prospective, randomized, open-label trials which were conducted from November 2008 to June 2009 in 15 emergency medical service (EMS) systems from 5 European countries [13] and from January 2010 to January 2018 in 11 EMS systems from 7 European countries [14]. Both trials tested the effectiveness and safety of pre-hospital intra-arrest trans-nasal evaporative cooling versus standard of care in non-traumatic OHCA patients with ongoing CPR. The inclusion criterion was bystander-witnessed cardiac arrest in patients at least 18 years of age. Some exclusion criteria differed between the two studies, in particular concerning the upper limit of age and the maximal EMS response time (i.e. from collapse to EMS arrival; Additional file 1: Table S1). Ethics and institutional committees in each participating country approved the study protocols, and written consent was obtained from closest relative or a legal representative of each patient after hospital admission and, at a later stage, from each patient who showed neurological recovery. Both studies were conducted according to the requirements of the Declaration of Helsinki. This pooled individual participant analysis was not pre-planned.

Patients were screened for eligibility by the EMS team after endotracheal intubation or supraglottic airway device placement and eventually randomized using sequentially numbered envelopes (1:1 allocation with different block designs) to receive either intra-arrest cooling using trans-nasal evaporative cooling or standard CPR. Both study groups received standard advanced life support treatment according to international guidelines, although the quality of CPR was not specifically monitored. For patients randomized to the trans-nasal evaporative cooling group, intra-arrest cooling was initiated using the RhinoChill device (BrainCool AB, Lund, Sweden), which delivers a mixture of air/oxygen and liquid cold evaporating perfluorohexane via nasal catheters, as previously described [13, 14]. In patients achieving ROSC or who were transported to the hospital with ongoing CPR, trans-nasal evaporative cooling was continued until systemic hospital cooling was initiated. Whole-body TTM was delivered in all admitted patients at target core temperature of 33 °C for at least 24 h, followed by a slow rewarming (0.2–0.5 °C per hour) and fever control for 72 h. Post-resuscitation care, including targets for ventilation settings, mean arterial blood pressure and glucose control were protocolized only in one study [14].

### Outcomes

Patients were followed up to hospital discharge [13] or to 90 days post-randomization [14]. For the purposes of this study, only patients randomized and eventually receiving the intervention were considered in the final analysis; as such, patients randomized to trans-nasal evaporative cooling and not receiving intra-arrest cooling or patients randomized to the control or the intervention group in whom CPR was immediately discontinued because of other reasons were excluded (i.e. “as-treated” analysis). This approach, which is “hypothesis generating” and associated with a post hoc analysis, has been already used in previous studies [15] and allow to investigate the effects of receiving the assigned treatment, as specified in the protocol, on patients’ outcome [16]. We excluded patients with missing information on primary outcome. Primary outcome in this pooled analysis was survival with favourable neurological outcome, defined as Cerebral Performance Category (CPC) scale score of 1–2, at hospital discharge. In both studies, EMS and hospital personnel were not blinded to treatment; however, neurological outcome was assessed by researchers who were blinded to the patients’ group assignments. Secondary outcomes included: survival at hospital discharge; ROSC rate; hospital admission rate; hospital and intensive care unit length of stay; complete neurological recovery (CPC 1) at hospital discharge. In particular, ROSC was defined as an organized rhythm and sustained palpable pulse that lasted at least 20 min in both studies. The intention-to-treat (ITT) analysis for neurological outcome was also reported.

### Data analyses and statistics

Variables from the two studies were both available from the original databases at the Karolinska Institutet in Stockholm (Sweden). Common variables between the two databases were pooled and included: patient demographics, baseline characteristics (including the use of bystander CPR and the initial rhythm) and main event times, such as time to EMS arrival, time to airway management, time to randomization, time to cooling (if in the intra-arrest cooling group), time to ROSC or CPR termination, time to defibrillation and doses of adrenaline or other drug. Clinical outcomes were harmonized into a single file, which was checked for accuracy of numbers, distributions and categories. Also, the last day of follow-up from arrest was recorded for each patient. For all analyses, no imputation was performed. We performed the analyses of primary and secondary outcomes in the group of patients according to their first cardiac rhythm. Thus, patients with an initial shockable rhythm and those with an initial non-shockable rhythm were presented separately. No specific adjustment was performed. The data were reported as relative risk (RR) and 95% confidence intervals (CIs). Additional analyses of the primary outcome were performed in the following subgroups: (a) age (≥ or < 80 years as this was the inclusion criterion of one study [14]); (b) EMS vs. helicopter-emergency medical system (HEMS), driven CPR (as HEMS-driven CPR was stopped because of prolonged time from arrest to inclusion [14]); (c) gender; (d) bystander CPR. All the statistical analyses for this study were processed in R version 3.6.0 (R Foundation for Statistical Computing, Vienna, Austria). P-values are two-tailed and values < 0.05 were considered statistically significant.

## Results

### Study population

**Fig. 1:**
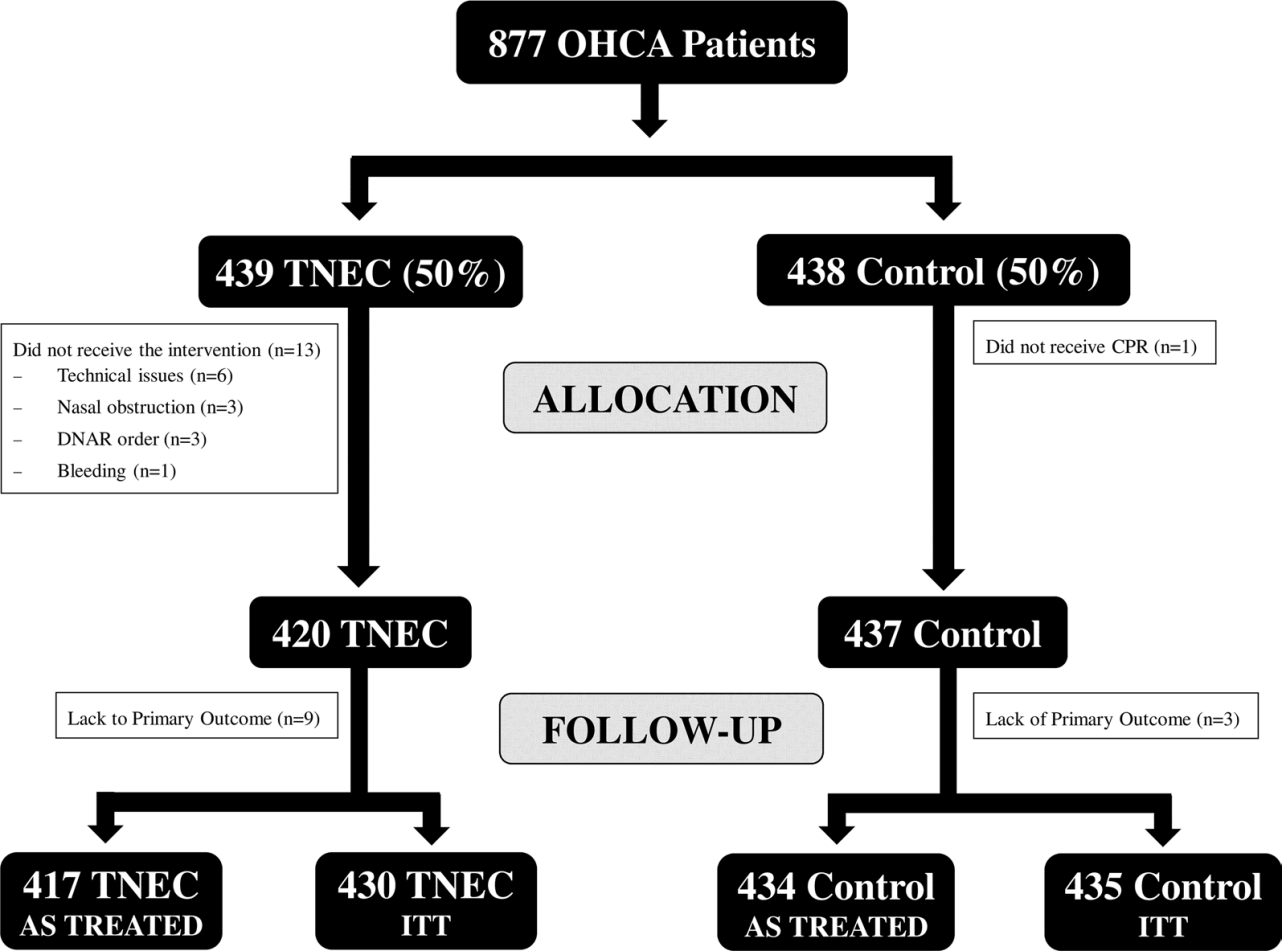
Flow chart of the subjects included in this analysis, according to the intention-to-treat (ITT) or “as-treated” approach. TNEC = trans-nasal evaporative cooling; CPR = cardiopulmonary resuscitation; DNAR = do not actively resuscitate

A total of 877 patients with witnessed OHCA were randomized in the two studies, of which 439 (50%) to the intervention group and 438 (50%) to the control group (Fig. 1). Of those, 12 patients had missing primary outcome data at hospital discharge (*n* = 9 intervention; *n* = 3 controls—i.e. 865 patients included in the ITT analysis, *n* = 430 in the intervention and *n* = 435 in the control group). Fourteen patients did not receive the assigned treatment; 13 in the intervention group, including 7 patients with a shockable rhythm (3 of them achieving CPC 1–2) and 6 with non-shockable rhythms (none with CPC 1–2) and 1 in the control group (CPC 5). A total of 851 (*n* = 417 in the intervention and *n* = 434 in the control group) patients were eligible for the final “as-treated” analyses. Of those, 325 (39%) had an initial shockable rhythm (158 in the intervention and 167 the control group), while 526 (62%) had an initial non-shockable rhythm (259 in the intervention and 267 in the control group). Main baseline characteristics are shown in Table 1. All variables were similar between the groups of patients with an initial shockable rhythm as within the groups of those with a non-shockable rhythm. However, patients with an initial shockable rhythm were younger, less likely to be men, had more frequently a cardiac cause of arrest and a bystander CPR than those with an initial non-shockable rhythm.

**Table 1:** Baseline characteristics of included patients with regard to the initial cardiac rhythm

	Shockable rhythms		Non-shockable rhythms	
	Intervention (*n* = 158)	Control (*n* = 167)	Intervention (*n* = 259)	Control (*n* = 267)
Age, years	64 [56–70]	62 [53–70]	67 [57–74]	67 [57–74]
Male gender, *n* (%)	143 (86)	130 (83)	174 (70)	187 (70)
Suspected cardiac origin of arrest, *n* (%)	147 (94)	136 (94)	189 (82)	189 (81)
Bystander CPR, *n* (%)	119 (74)	113 (75)	122 (46)	122 (50)
Estimated time from arrest to CPR (min)	8 [6–11]	9 [6–12]	8 [5–11]	8 [6–12]
Estimated time from call to CPR (min)	7 [5–10]	7 [5–10]	7 [5–10]	8 [5–12]
Estimated time from arrest to ALS (min)	13 [9–17]	12 [9–17]	13 [9–16]	12 [9–17]
Estimated time from arrest to airway protection (min)	15 [11–20]	14 [10–18]	14 [10–18]	14 [11–18]
Estimated time from arrest to randomization (min)	18 [13–23]	17 [13–21]	18 [13–23]	16 [12–21]
Estimated time from arrest to ROSC (min)	30 [19–40]	25 [20–33]	30 [26–37]	30 [20–41]
Estimated time from arrest to hospital admission (min)	52 [44–62]	52 [38–62]	53 [44–65]	55 [42–70]
Estimated time from arrest to hospital cooling (min)	102 [80–151]	93 [64–151]	97 [71–137]	103 [82–129]
Number	98	99	80	84
Temperature at ER—tympanic, mean (SD)	34.87 (1.15)^a^	35.76 (0.80)	34.42 (1.40)^a^	35.60 (0.72)
Temperature at ICU—tympanic, mean (SD)	34.82 (1.16)^a^	35.38 (0.96)	34.45 (1.32)^a^	35.55 (0.92)
Temperature at ICU—core, mean (SD)	34.87 (0.96)^a^	35.33 (0.90)	34.47 (1.38)^a^	35.35 (1.06)

Data are presented as count (%) median [IQRs] or mean (SD)

*CPR* cardiopulmonary resuscitation, *ROSC* return of spontaneous circulation, *ALS* advanced life support, *ER* emergency room, *ICU* intensive care unit

^a^*p* value < 0.05 between intervention and control group

### Outcomes in all patients

**Fig. 2:**
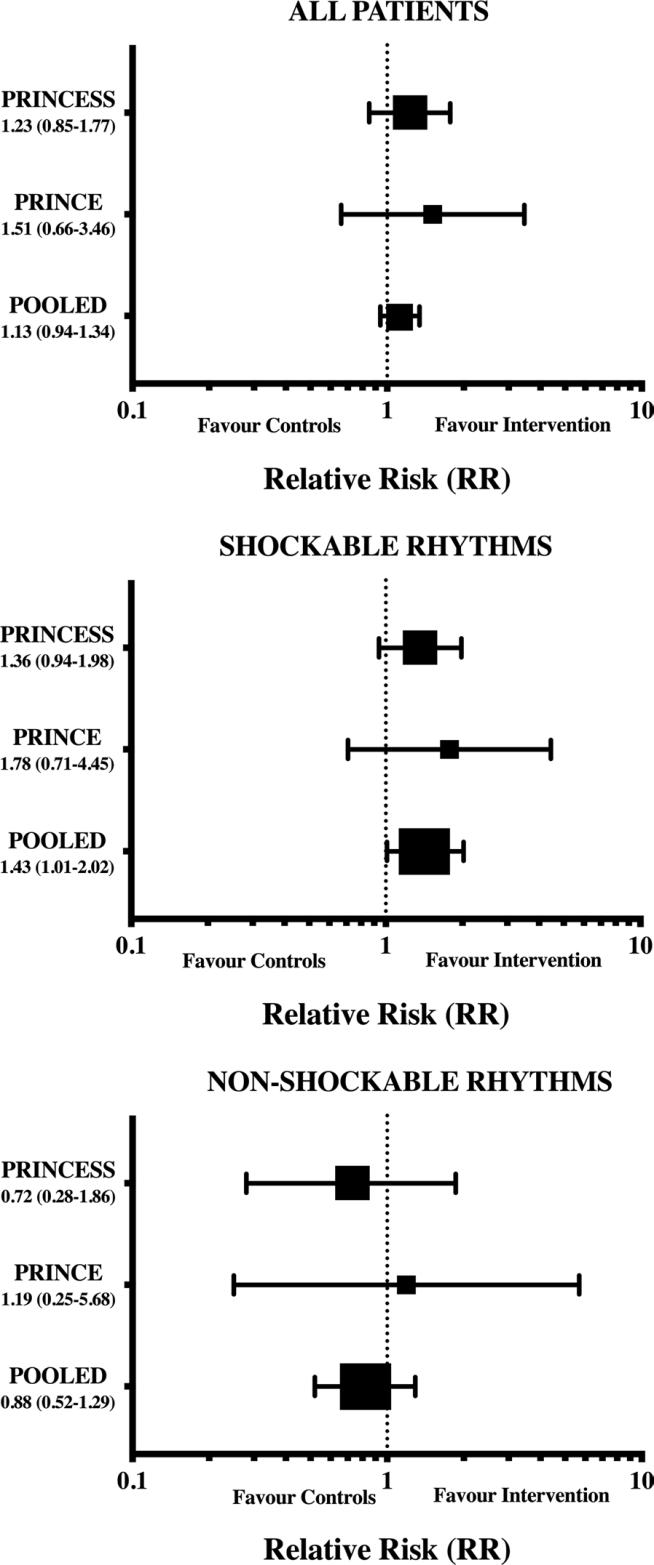
Pooled analyses of favourable neurological outcome (CPC 1–2) at hospital discharge in all included patients and in the subgroup of patients with shockable and non-shockable rhythm. PRINCE (13) and PRINCESS (14)

Outcome analyses for all included patients are presented in Fig. 2 and Additional file 2: Fig. S1. The number of patients who survived with favourable neurologic outcome at hospital discharge was 64/417 (15.3%) in the intervention and 53/434 (12.2%) in the control group (RR 1.13 [CIs 0.94–1.34]; *p* = 0.19). Survival to hospital discharge was observed in 77/417 (18.4%) patients in the intervention and in 68/434 (15.6%) in the control group (RR 1.10 [CIs 0.92–1.30]; *p* = 0.31—Additional file 2: Fig. S1).

### Outcomes in patients with initial shockable rhythms

In the primary outcome analysis, the number of patients who survived with favourable neurologic outcome at hospital discharge was 54/158 (34.2%) in the intervention and 40/167 (24.0%) in the control group (RR 1.43 [CIs 1.01–2.02]; *p* = 0.049—Table 2, Fig. 2). Survival to hospital discharge was observed in 63/158 (39.9%) patients in the intervention and in 51/167 (30.5%) in the control group (RR 1.31 [CIs 0.97–1.76]; *p* = 0.06—Table 2, Additional file 2: Fig. S1).

**Table 2:** Main outcomes with regard to the initial cardiac rhythm

	Shockable rhythms		Non-shockable rhythms	
	Intervention (*n* = 158)	Control (*n* = 167)	Intervention (*n* = 250)	Control (*n* = 267)
*Primary outcome*				
Survival with favourable neurological outcome at hospital discharge [*n* (%)]	54 (34.2)	40 (24.0)^a^	10 (4.0)	13 (4.9)
*Secondary outcomes*				
Survival at hospital discharge [*n* (%)]	63 (39.9)	51 (30.5)	14 (5.6)	17 (6.4)
ROSC on scene [*n* (%)]	100 (63.2)	110 (65.8)	99 (39.6)	102 (38.2)
Hospital admission [*n* (%)]	98 (62.0)	99 (59.3)	78 (31.2)	84 (31.5)
ICU length of stay (days)	4 [2–8]	3 [1–7]^a^	3 [1–5]	2 [1–5]
Days on mechanical ventilation	3 [2–5]	3 [1–5]	2 [1–4]	2 [1–5]
Hospital length of stay (days)	6 [2–14]	5 [2–15]^a^	3 [1–5]	2 [1–5]
Complete neurological recovery [*n* (%)]	40 (25.3)	27 (16.2)^a^	4 (1.6)	5 (1.9)

Data are presented as count (%) or median [IQRs]

^a^*p* value < 0.05 between intervention and control group

**Fig. 3:**
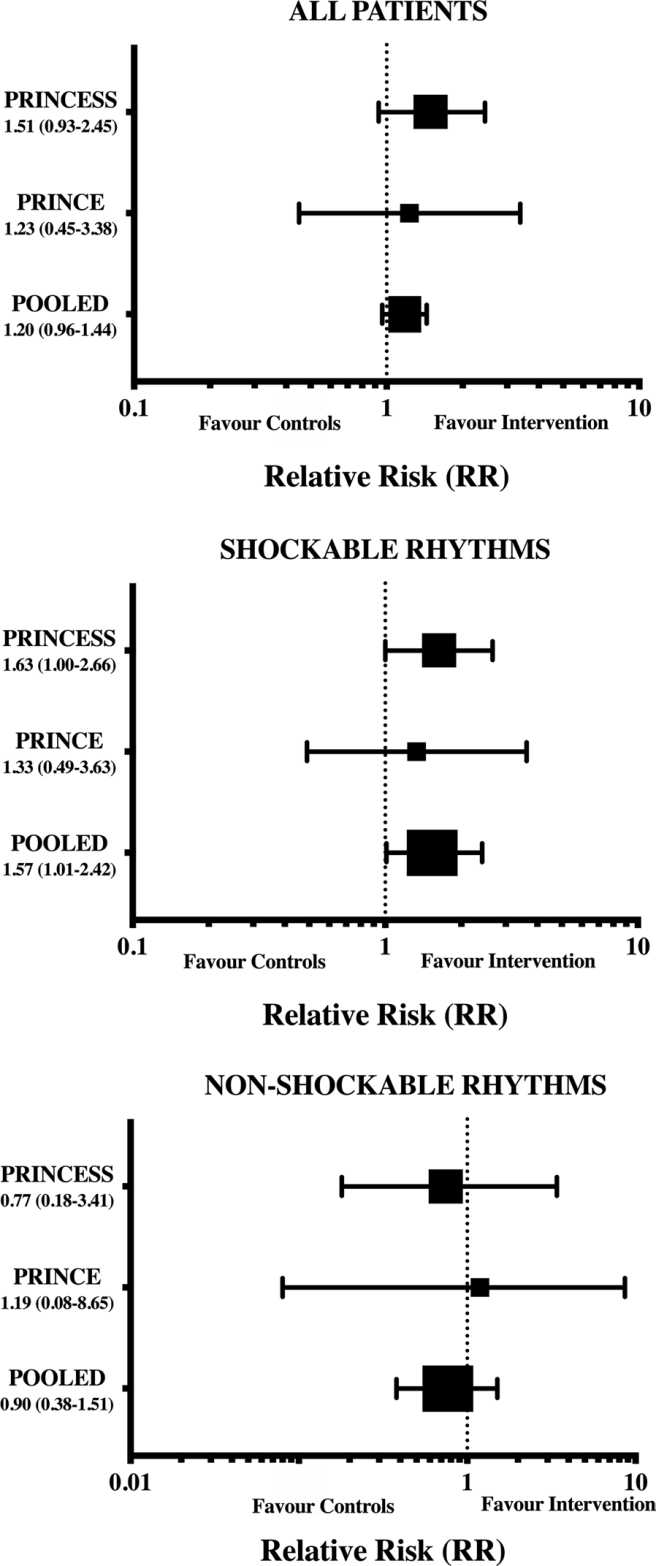
Pooled analyses of complete neurological outcome (CPC 1) at hospital discharge in all included patients and in the subgroup of patients with shockable and non-shockable rhythm. PRINCE (13) and PRINCESS (14)

In the secondary outcome analysis, complete neurological recovery was observed in 40/158 (25.3%) patients in the intervention and in 27/167 (16.2%) in the control group (RR 1.57 [CIs 1.01–2.42]; *p* = 0.04—Table 2, Fig. 3). No differences in ROSC (RR: 0.94 [CIs 0.75–1.19]; *p* = 0.64) and hospital admission rates (RR: 1.06 [CIs 0.85–1.35]; *p* = 0.65) were observed between groups. Distribution of CPC Scores at hospital discharge is presented in the Additional file 3: Fig. S2.

### Outcomes in patients with initial non-shockable rhythms

In the primary outcome analysis, the number of patients who survived with favourable neurologic outcome at hospital discharge was 10/259 (3.8%) in the intervention and 13/267 (4.9%) in the control group (RR 0.88 [CIs 0.52–1.29]; *p* = 0.67—Table 2, Fig. 2). Also, survival at hospital discharge was observed in 14/259 (5.4%) patients in the intervention and in 17/267 (6.4%) in the control group (RR 0.91 [CIs 0.59–1.28]; *p* = 0.71—Table 2, Additional file 2: Fig. S1).

In the secondary outcome analysis, complete neurological recovery was observed in 4 (1.5%) patients in the intervention and in 5 (1.9%) in the control group (RR 0.90 [CIs 0.38–1.51]; *p* = 0.98—Table 2, Fig. 3). No differences in ROSC (RR: 1.03 [CIs 0.86–1.23]; *p* = 0.79) and hospital admission rates (RR: 0.97 [CIs 0.79–1.16]; *p* = 0.77) were observed between groups. The interaction of the intervention between the two groups (i.e. shockable vs. non-shockable rhythm) was not significant (*p* = 0.11).

### Outcomes in all patients with ITT analysis

Neurological outcome analyses for all patients according to the ITT approach are presented in Additional file 5: Fig. S4. The number of patients who survived with favourable neurologic outcome at hospital discharge was 67/430 (15.5%) in the intervention and 53/434 (12.2%) in the control group (RR 1.23 [CIs 1.01–1.44]; *p* = 0.039). Among patients with an initial shockable rhythm, the number of patients who survived with favourable neurologic outcome at hospital discharge was 56/165 (33.9%) in the intervention and 40/167 (23.9%) in the control group (RR 1.26 [CIs 1.00–1.56]; *p* = 0.052). Among patients with an initial non-shockable rhythm, the number of patients who survived with favourable neurologic outcome at hospital discharge was 11/265 (4.1%) in the intervention and 13/267 (4.8%) in the control group (RR 0.91 [CIs 0.55–1.31]; *p* = 0.83).

### Additional analyses

Among patients with a shockable rhythm and EMS-treated (in opposite to HEMS-treated), 51/140 (36.4%) in the intervention and 35/148 (23.6%) in the control group had CPC 1–2 at hospital discharge (RR 1.54 [CIs 1.07–2.21]; *p* = 0.02—Additional file 4: Fig. S3). Among female patients with a shockable rhythm, 13/27 (48.1%) in the intervention and 4/23 (17.4%) in the control group had CPC 1–2 at hospital discharge (RR 2.77 [CIs 1.05–7.32]; *p* = 0.03—Additional file 1: Fig. S1). Additional subgroup analyses did not show any statistical differences (Additional file 4: Fig. S3).

## Discussion

In this pooled analysis of individual patient level data obtained from two randomized studies on the use of intra-arrest trans-nasal evaporative cooling in OHCA patients at the scene of the arrest compared to TTM initiated after hospital arrival, we showed that the intra-arrest cooling was associated with a significantly higher proportion of favourable neurological outcome at hospital discharge in patients with an initial shockable rhythm. In addition, the proportion of patients with complete neurological recovery was higher in the intra-arrest cooling than in the control group. No differences in ROSC rate and hospital admission were observed between groups. No difference in any outcome measure was observed in patients with non-shockable rhythms and when the entire cohort was analysed.

The main finding from this analysis was the benefits of intra-arrest cooling in patients with an initial shockable rhythm. In the previous studies, results suggesting an effect of intra-arrest cooling on functional neurological recovery were obtained only in *post hoc* analyses of patients admitted to the hospital [13] or in a pre-planned subgroup analysis of all randomized patients [14]. The large effects of cooling procedures in this subgroup of patients with shockable rhythms were also observed in the first randomized studies comparing TTM at hospital to standard of care [4, 5] as well as in subgroup analysis on prolonged TTM for 48 h when compared to standard 24-h duration, with the core body temperature maintained of 33 °C [17]. Although a recent randomized study also showed that TTM was effective among selected comatose survivors after cardiac arrest with non-shockable rhythm when compared to targeted normothermia [7], no effect of intra-arrest cooling was observed in patients with non-shockable rhythms in this pooled analysis. The different effects of intra-arrest cooling on shockable and non-shockable rhythms could not be explained by difference in the intervention times nor by a longer time to cooling, as these variables were similar between groups. Nevertheless, patients with an initial non-shockable rhythm were older, had more frequently a non-cardiac cause of arrest and less bystander CPR than those with shockable rhythm, and all these factors are independently associated with poor outcome after cardiac arrest [18–20] and may have blunted the potential benefits of the intervention. We observed a non-significant difference between subgroup-specific subgroups when a formal test of interaction was performed; although this might be related to an underpowered sample size, this finding can also suggest that other confounders might influence such results, which should therefore be considered as mostly exploratory. Moreover, the data on shockable rhythm suggest some “imprecision”, as the CIs are very close to 1 and results are not consistent with the ITT analysis. Future randomized trials including larger cohorts of OHCA patients should therefore target shockable rhythm as the population who might benefit the most from intra-arrest cooling with the use of trans-nasal evaporative cooling devices.

The most common limitations in applying intra-arrest cooling for OHCA are the need for a standardized protocol, the lack of an effective TTM device and the long time from arrest to cooling initiation. Pre-hospital care of OHCA patients should primarily focus on the early initiation of high-quality CPR and, for patients with shockable rhythms, on early defibrillation [21]. As such, the implementation of an additional intervention such as intra-arrest cooling could perhaps negatively impact on the quality of CPR and on standardized resuscitation protocols. However, we did not observe differences in ROSC rates nor in the proportion of patients admitted to the hospital when the intra-arrest cooling group and the control group were compared. In experimental studies, trans-nasal cooling initiated at the start of CPR significantly improved the success of defibrillation and ROSC rates in a prolonged (15 min) cardiac arrest model [22]. Although these results were never replicated in the human setting, we did not observe any safety issue on the effectiveness of CPR when intra-arrest cooling was implemented. Concerning the optimal method to deliver intra-arrest TTM, animal studies showed that the use of total lung ventilation or trans-nasal evaporative cooling was associated with a higher ROSC rate than cold fluids, although no difference in the survival rate was found [23, 24]. In a large study (*n* = 1198), patients with an initial shockable rhythm and treated with intra-arrest cooling using cold fluids had a lower ROSC rate than the standard group (41% vs. 51%, *p* = 0.03) [9]. The reduction of the coronary perfusion pressure as well as pulmonary overload might explain such deleterious effects observed with cold fluids [24]. No benefits were observed in another RCT for intra-arrest cold fluids when compared to standard TTM [25]. Trans-nasal evaporative cooling has been developed to primarily cool the brain and has not been associated with any deleterious hemodynamic effect and should be considered as the best available method in this setting.

Some questions regarding trans-nasal evaporative cooling remain: the time from arrest to cooling initiation remained long in this study; airway protection was mandatory before randomization because of the unknown effects on systemic oxygenation resulting from the alveolar filling of cold evaporative coolant. In one recent study, trans-nasal evaporative cooling initiated before achieving a protected airway during CPR in the prehospital setting was not associated with any specific side effects than epistaxis or nose whitening [26]. As such, future studies should consider early application (i.e. immediately after EMS arrival and before intubation) of intra-arrest trans-nasal evaporative cooling in OHCA patients to reduce the time to selective brain cooling and to increase the potential protective effects of this intervention. Moreover, feasibility of a study protocol including intra-arrest cooling using TNEC could be questionable, as the PRINCESS study [14] required almost 8 years to be completed; as such, a large collaborative research network will be necessary to eventually complete future large RCTs on this topic in a reasonable timeframe.

The strengths of these analyses include the relatively large dataset derived from patients recruited from several European centers, with systematic evaluations of neurological outcome and few losses of follow-up. Considering the difficulties in completing this type of study, this analysis could provide relevant information in a specific patient population (i.e. initial shockable rhythm) in whom a trend towards better outcome was observed in the two previous RCTs, which were somewhat underpowered to specifically assess this issue [13, 14]. Moreover, the effects on neurological outcome were mainly driven by a higher number of patients with complete neurological recovery, which would suggest a strong neuroprotective effect of intra-arrest cooling. Finally, the number of patients to treat to obtain one additional patient with a favourable neurological outcome was 10, which was consistent with the large absolute improvement in neurological recovery observed in the primary outcome analysis.

However, there are several limitations to this study that need to be considered when interpreting the findings. First, extrapolation of our findings to all EMS settings should be done with caution as participating centers were experienced with the use of the device and only European EMS were included. Second, the quality of CPR was not specifically assessed. However, we did not observe any delay in the time to the initiation of chest compression, time to defibrillation and other advanced life support interventions. Third, follow-up was limited to hospital discharge as long-term outcome data were not available in one of the two studies [13]. Most of recent randomized studies conducted in OHCA patients collected data at 3 or 6 months after randomization [7, 14, 15]; one may argue that early assessment of neurological outcome would be limited as global measures of cerebral disability indicated recovery between one month and one year after cardiac arrest [27]. Also, recent consensus suggested to use the modified Rankin scale score rather than CPC scale to assess neurological outcome after cardiac arrest, together with different testing of quality of life and cognitive dysfunction [28], which were not available in our database. Fourth, neurological prognostication followed a common protocol, although this was not entirely standardized between the two studies. As such, withdrawal of life-sustaining therapies policy might have been different between groups. Fifth, we excluded patients who did not receive the intervention as assigned to minimize bias due to the lack of cooling exposure or early termination of resuscitation, despite these patients were part of the intention to treat analysis for both studies. This analysis approach was considered the most appropriate to assess the effectiveness of the intervention according to patients’ exposure. However, the results of this analysis remain “hypothesis generating” and future studies evaluating the effects of trans-nasal evaporative cooling in OHCA patients with an initial shockable rhythm are needed to confirm its potential effectiveness. Fifth, this pooled individual participant analysis was not pre-planned and the decision to perform such post hoc study was based on the results of the PRINCESS study, suggesting again a potential benefit of the intervention for patients with shockable rhythms. Sixth, intra-arrest hypothermia using cold fluids has been associated with a significant increased risk of re-arrest, even when given after ROSC [9, 29]; however, these two complications were not specifically reported in the PRINCE and PRINCESS trials. Finally, the time from arrest to hospital cooling was quite short (i.e. a median 103 min in the longest subgroup), which is not reproducible in all hospital settings and might limit the generalizability of these findings.

## Conclusions

In this pooled analysis of individual participant data, the use of intra-arrest evaporative cooling was associated with a significant increase in favourable neurological recovery at hospital discharge among OHCA patients with an initial shockable rhythm. The intervention was also associated with a significant increase in completed neurological recovery at hospital discharge among those patients. No outcome differences were observed in patients with non-shockable rhythms.

## Supplementary Information

**Additional file 1. Table S1**: Main differences in inclusion and exclusion criteria between the two studies.

**Additional file 2. Fig. S1**: Pooled analyses of survival at hospital discharge in all included patients and in the subgroup of patients with shockable and non-shockable rhythm. PRINCE [13] and PRINCESS [14].

**Additional file 3. Fig. S2**: Distribution of Cerebral Performance Category (CPC) scores at hospital discharge after cardiac arrest according to the initial rhythm.

**Additional file 4. Fig. S3**: Patients with Cerebral Performance Category (CPC) 1–2 at hospital discharge after cardiac arrest according to subgroup analyses.

**Additional file 5. Fig. S4**: Pooled analyses of favourable neurological outcome (CPC 1–2) at hospital discharge in all included patients and in the subgroup of patients with shockable and non-shockable rhythm according to the intention-to-treat (ITT analysis).

## Data Availability

All data generated or analysed during this study are included in this published article (and its supplementary information files).

## Abbreviations

CA: Cardiac arrest
CIs: Confidence intervals
CPC: Cerebral performance category
CPR: Cardiopulmonary resuscitation
EMS: Emergency medical service
HEMS: Helicopter emergency medical service
OHCA: Out-of-hospital cardiac arrest
RCTs: Randomized controlled trials
ROSC: Return of spontaneous circulation
RR: Relative risk
TTM: Targeted temperature management
