# Supplementary material for: Effect of intra-arrest trans-nasal evaporative cooling in out-of-hospital cardiac arrest: a pooled individual participant data analysis
Source: Crit Care. 2021 Jun 8;25:198. doi: 10.1186/s13054-021-03583-9 (PMC8188685; doi:10.1186/s13054-021-03583-9)
Supplement: Supplementary file 1 — Additional file 1. Table S1: Main differences in inclusion and exclusion criteria between the two studies. [file 13054_2021_3583_MOESM1_ESM.docx]

**Supplemental Table 1.** Main differences in inclusion and exclusion criteria between the two studies.

|  | **PRINCE [13]** | **PRINCESS [14]** |
| --- | --- | --- |
| **Inclusion Criteria** | | |
| Age | > 18 years | > 18 and < 80 years |
| ROSC prior to randomization | No | No |
| Witnessed CA | Yes | Yes |
| Time from arrest to EMS arrival | < 20 minutes | < 15 minutes |
|  |  |  |
| **Exclusion Criteria** | | |
| Non-cardiac cause of arrest | Yes | Yes |
| Known Oxygen-Dependency | Yes | Yes |
| Known Coagulation Disorders | No | Yes |
| Do-Not-Resuscitation Order | Yes | Yes |
| Terminal Illness | Yes | Yes |
| Nasal Obstruction | Yes | Yes |
| Already Hypothermic | No | Yes |

ROSC = return of spontaneous circulation; CA = cardiac arrest; EMS = Emergency Medical System
