# Supplementary figures and images for: Effect of intra-arrest trans-nasal evaporative cooling in out-of-hospital cardiac arrest: a pooled individual participant data analysis
Source: Crit Care. 2021 Jun 8;25:198. doi: 10.1186/s13054-021-03583-9 (PMC8188685; doi:10.1186/s13054-021-03583-9)

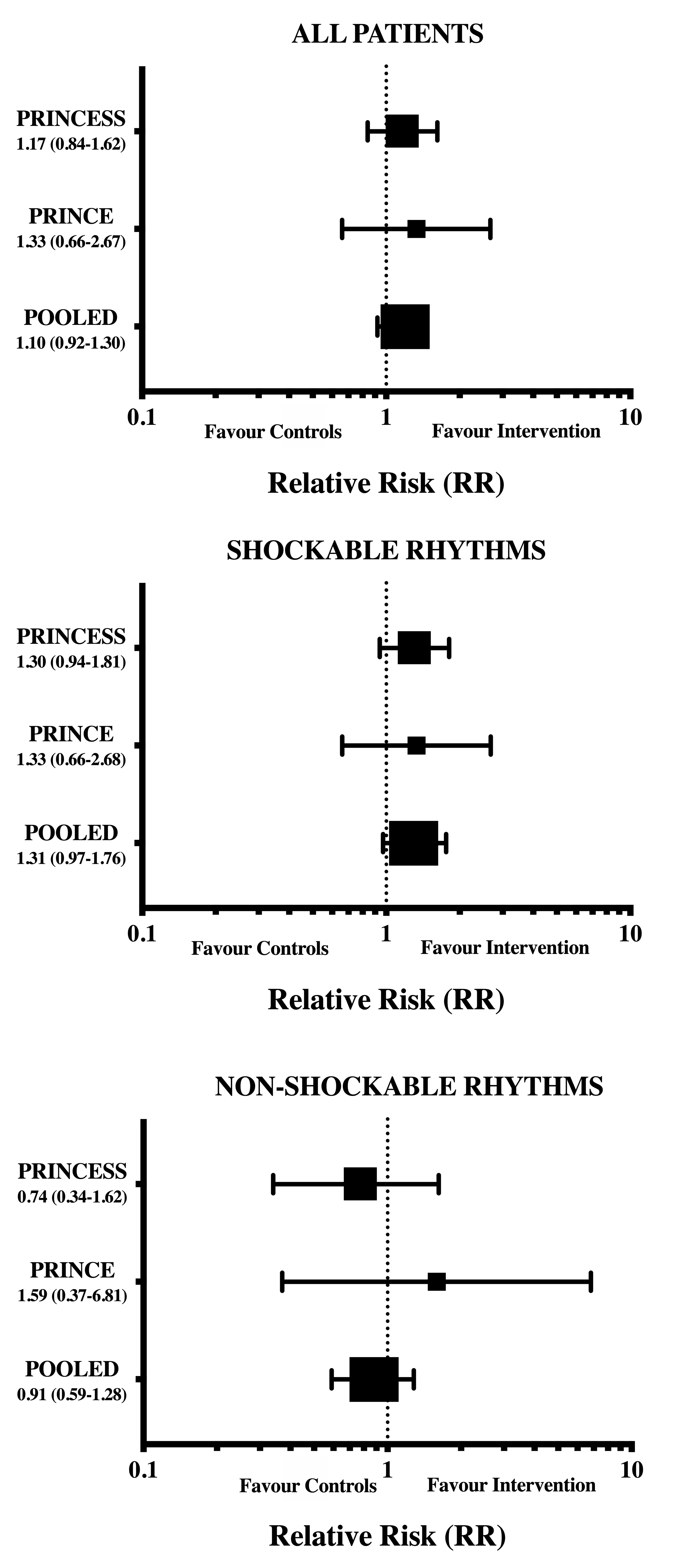

Supplement: Supplementary file 2 — Additional file 2. Fig. S1: Pooled analyses of survival at hospital discharge in all included patients and in the subgroup of patients with shockable and non-shockable rhythm. PRINCE [13] and PRINCESS [14]. [file 13054_2021_3583_MOESM2_ESM.tiff]

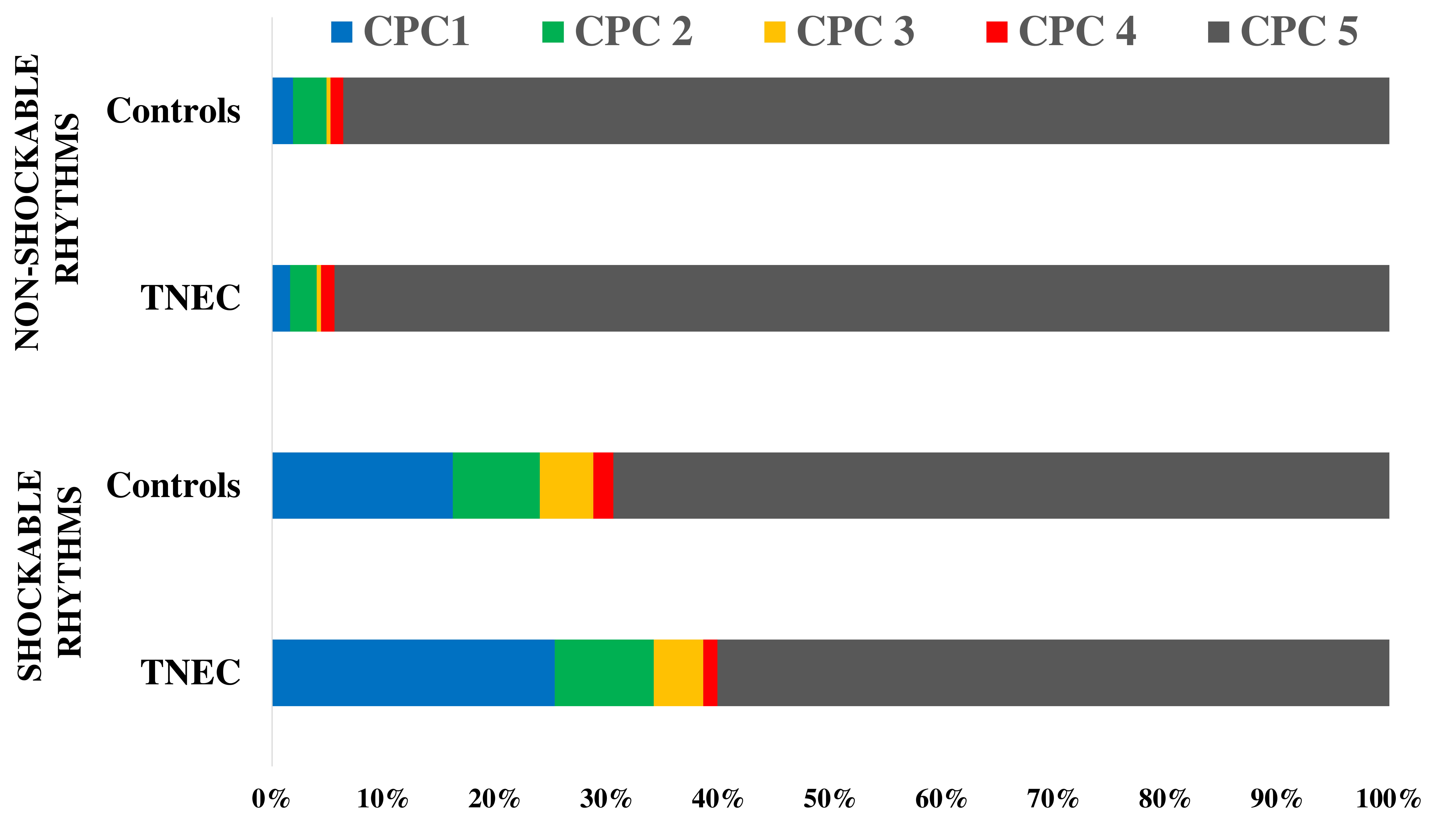

Supplement: Supplementary file 3 — Additional file 3. Fig. S2: Distribution of Cerebral Performance Category (CPC) scores at hospital discharge after cardiac arrest according to the initial rhythm. [file 13054_2021_3583_MOESM3_ESM.tiff]

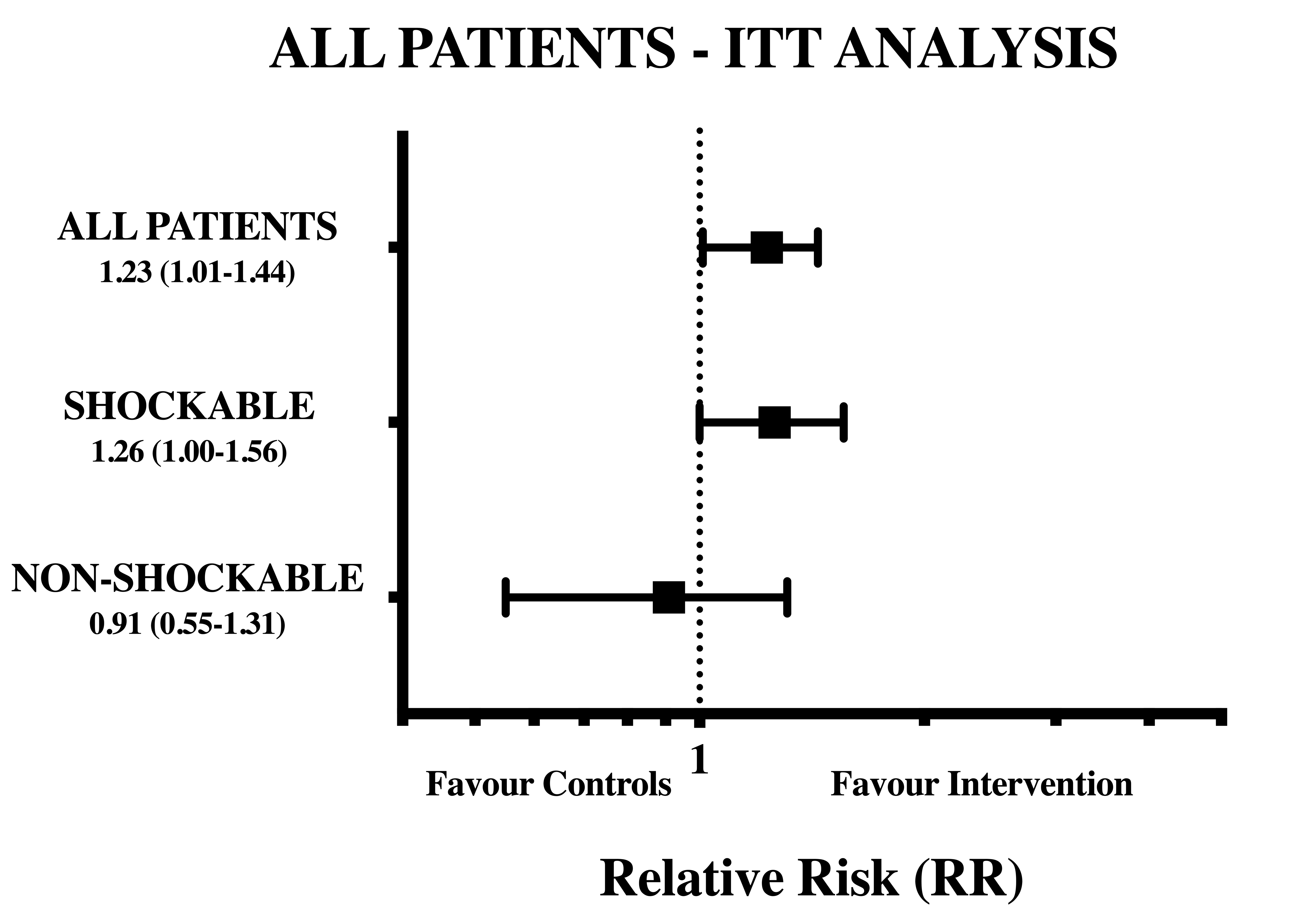

Supplement: Supplementary file 5 — Additional file 5. Fig. S4: Pooled analyses of favourable neurological outcome (CPC 1–2) at hospital discharge in all included patients and in the subgroup of patients with shockable and non-shockable rhythm according to the intention-to-treat (ITT analysis). [file 13054_2021_3583_MOESM5_ESM.tiff]
