# Supplementary material for: Effect of intra-arrest trans-nasal evaporative cooling in out-of-hospital cardiac arrest: a pooled individual participant data analysis
Source: Crit Care. 2021 Jun 8;25:198. doi: 10.1186/s13054-021-03583-9 (PMC8188685; doi:10.1186/s13054-021-03583-9)

**Supplemental Figure 3:** Patients with Cerebral Performance Category (CPC) 1-2 at hospital discharge after cardiac arrest according to subgroup analyses.


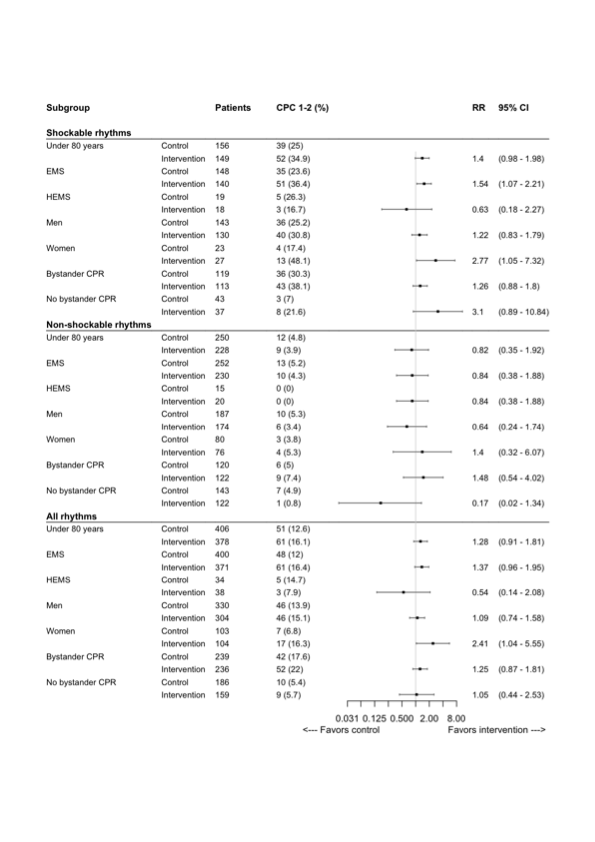

Supplement: Supplementary file 4 — Additional file 4. Fig. S3: Patients with Cerebral Performance Category (CPC) 1–2 at hospital discharge after cardiac arrest according to subgroup analyses. [file 13054_2021_3583_MOESM4_ESM.docx]
